# Knights in shining armour and (M)others in life jackets: Women’s experiences of advocating for care alone when suffering recurrent early pregnancy loss during the SARS-CoV-2 pandemic

**DOI:** 10.1186/s12889-024-20882-2

**Published:** 2025-01-29

**Authors:** Flora E. Kent-Nye, Kayleigh S. Sheen, Karen Burgess, Munira Oza, Laura A. Magee, Davor Jurković, Sergio A. Silverio

**Affiliations:** 1 Department of Women & Children’s Health, King’s College London, London, UK; 2 Department of Social Sciences, University of the West of England Bristol, Bristol, UK; 3 PETALS: The Baby Loss Counselling Charity, Cambridge, UK; 4 The Ectopic Pregnancy Trust, London, UK; 5 Gynaecology Diagnostic and Treatment Unit, University College London Hospitals NHS Foundation Trust, London, UK; 6 Department of Psychology, University of Liverpool, Liverpool, UK

**Keywords:** Early pregnancy, Recurrent pregnancy loss, Early pregnancy assessment unit, Emotions, Pregnancy loss, Qualitative research, Grounded theory, COVID-19, SARS-CoV-2

## Abstract

**Background:**

Recurrent early pregnancy loss [rEPL] is a traumatic experience, marked by feelings such as grief and depression, and often anxiety. Despite this, the psychological consequences of rEPL are often overlooked, particularly when considering future reproductive health or approaching subsequent pregnancies. The SARS-CoV-2 pandemic led to significant reconfiguration of maternity care and a negative impact on the perinatal experience, but the specific impact on women’s experience of rEPL has yet to be explored. This study aimed to examine the impact of changes to early pregnancy loss care and social restrictions during the pandemic on women’s experience of rEPL.

**Methods:**

A qualitative interview study design was employed, with semi-structured interviews conducted virtually. A total of 16 women who had suffered two or more early pregnancy losses (≤ 14 weeks gestation) during the SARS-CoV-2 pandemic in the United Kingdom participated. Data were recorded, transcribed, and analysed by hand, following a Classical Grounded Theory Analysis, appropriate for cross-disciplinary health research.

**Results:**

Iterative and inductive analysis generated the theory ‘Knights in Shining Armour and (M)others in Life Jackets’, which describes women’s experience of advocating for care alone, when suffering rEPL during the pandemic. This theory was derived from the way in which three emergent themes inter-related: (1) Dismantling Validation; (2) Preserving an Identity of Motherhood; and (3) Support Waning.

**Conclusions:**

This study affirms recent findings of devalued maternity care during the pandemic, and magnification of pre-pandemic issues with EPL care, such as a lack of support or perceived empathy.

**Supplementary Information:**

The online version contains supplementary material available at 10.1186/s12889-024-20882-2.

## Background

Early pregnancy loss [EPL] can represent one of the most traumatic health-related events across a woman’s lifecourse. EPL is common, and most frequently due to miscarriage, but may also result from ectopic pregnancy, molar pregnancy, early elective abortion, or pregnancy of unknown location (see Table 1). There is considerable debate about what constitutes ‘recurrent’ early pregnancy loss [rEPL], particularly with regards to the number of losses required and whether these need to be consecutive [1–4]. Latest guidance from the European Society of Human Reproduction and Embryology [ESHRE] Recurrent Pregnancy Loss Guideline Development Group [5] defines recurrent pregnancy loss as more than two, whereas the Royal College of Obstetricians and Gynaecologists [RCOG] suggests recurrent is defined as three or more first trimester miscarriages [6]. Both guidelines, however, suggest clinical judgement and suspected pathological cause are reason enough to commence earlier investigation.

**Table 1:** Definitions and UK Incidence Rates of early pregnancy loss

Type of Pregnancy Loss	Definition	Approximate UK Incidence Rate (out of clinically recognised pregnancies)
Early Miscarriage (incl. Chemical Pregnancy)	A spontaneous loss of a pregnancy occurring within the first twelve weeks of gestation.	1 in 4 [7]
Pregnancy of Unknown Location [PUL]	Where there is a positive human chorionic gonadotrophin (hCG) urine test but with no signs of pregnancy on ultrasound scan in a patient who is otherwise well.	1 in 10 [8]
Termination of Pregnancy [ToP]	The termination of a pregnancy within the first trimester.	1 in 54 [9]
Ectopic Pregnancy	When a fertilised egg implants itself outside of the uterine cavity, usually in one of the fallopian tubes.	1 in 100 [10]
Molar Pregnancy (Gestational Trophoblastic Disease)	Where fertilisation does not occur correctly, and the result is an abnormal non-viable pregnancy/growth of cells, sometimes resembling clusters of grapes inside the womb.	1 in 590 [11]

EPL exacts a physical and emotional toll on women which is frequently under-reported and unrecognised [12]. In addition to the pain suffered from the pregnancy loss, the psychological impact of EPL can evoke a broad range of feelings, from shock, guilt, sadness, grief, and frustration, to more severe, clinical episodes of anxiety and depression – all unique to a woman’s circumstances [12–16]. Often EPL is abrupt, causing women to lurch from one psycho-social state to another – from expectant to bereaved mother [17, 18], which may challenge individuals’ identity as mothers and as women [17, 19]. These responses may be persistent, with long-term implications for mental health in subsequent pregnancies [20], particularly if a woman has no living children [21] or if EPL is recurrent [22, 23].

Due to the frequency of EPL, most women will refrain from disclosing their pregnancy early-on [24]. During this period of concealment, many expectant mothers may already acclimatise to the idea of motherhood and begin forming an emotional bond to their baby [25]. In the event of an EPL, this can result in disenfranchised grief, i.e., the loss of a loved one not publicly mourned or acknowledged due to a lack of social recognition [26]. Many bereaved mothers therefore struggle to allow themselves to grieve, deny the depth of their pain, or suffer in public, silently; with grief becoming intrusive, disruptive, and long-lasting [27, 28]. Despite this, women often receive little-to-no formal psychological support [15].

In response to the unique clinical care needs of women with EPL, the UK has developed dedicated Early Pregnancy Assessment Units [EPAUs], where women with EPL can receive comprehensive care, by dedicated, expert staff [15, 29–34]. The impact of the pandemic and pandemic-related healthcare service reconfigurations on perinatal care, including care for perinatal bereavement, has documented devalued care and poor psycho-social outcomes [35–44]. However, the impact of the pandemic on women with rEPL has not previously been studied.

## Methods

### Aim and the present study

The aim of this qualitative Grounded Theory interview study was to explore the impact of changes to maternity care and social restrictions during the SARS-CoV-2 pandemic on women’s experience of rEPL.

The study team was multidisciplinary, comprising psychologists [SAS, KSS], a gynaecologist [DJ], an obstetric physician [LAM], those working in the charitable sector dedicated to pregnancy loss [KB, MO], and a female student researcher with a background in medical physiology [FEK-N] who led the interviews. This project followed protocols for best practice in qualitative research into sensitive, challenging, and difficult topics [45].

### Ethics

Ethical approvals were granted by the King’s College London Health Faculties Research Ethics Sub-committee (ref:-HR/DP-21/22-28808). All participants provided consent to participate. For full details on study ethics and recruiting procedure, please refer to a prior publication from this study [44].

### Theoretical perspective

We adopted a gendered lifecourse analysis approach [46]. We positioned pregnancy and childbirth as part of the normative lifecourse for women in a Western setting (such as the UK); as such, a pregnancy loss presents as a lifecourse rupture which results in a significant adjustment in the childbearing and mothering trajectory, and offers a site for empirical inquiry [17]. To this end, our research paradigm was post-positivist, whereby participants’ narratives were accepted as ‘truths’ or ‘lived realities’ [47].

Given the adoption of a post-positivist paradigm [48], the study was philosophically underpinned by ontological critical realism [49] and an objectivist epistemology [47]. This allowed us to interpret the lived realities relayed to us in interviews through a lens which recognized both social contexts and conditioning [50], and that truth can be acquired even with the acquisition of fallible knowledge [51].

We adopted an empathic, yet critical reflexive judgement of interview data [52], meaning we appraised our participants’ narratives both with a level of criticality, and an understanding of the differing reactions women may have to rEPL and the wider context of the SARS-CoV-2 pandemic. We acknowledge the production of knowledge is entrenched within one’s socio-political and cultural background, accepting that complete objectivity is impossible, and that understanding the value we might attribute experiences is laden in the context of changing structural conditions and social pressures.

Our position within the data was as a combination of objective outsiders and subjective spectators [53], due to the mix of lived experience of suffering rEPL or working in the field, clinically or academically. Furthermore, in an attempt to set aside any unacknowledged preconceptions related to the research, bracketing [54] was utilized, whereby we attempted to set aside most of one’s personal suppositions and holding them in abeyance, but acknowledging them in the interpretive phases of the project.

### Recruitment, setting, and participants

Women were recruited on-line, using social media, charitable partners, and snowballing to disseminate advertisements. Women were eligible to participate if they: (1) were over the age of 18 years; (2) had experienced one or more EPL (≤ 14 weeks’ gestation); and (3) those EPLs had been during the SARS-CoV-2 pandemic (since 30 January 2020). For this analysis, only women who experienced two or more pregnancy losses during the pandemic were included.

Theoretical sampling was undertaken, purposefully sampling participants with similar characteristics to those demonstrating narrative anomalies in the dataset. This investigated whether these individuals were a distinct group that should be analysed separately, or whether their responses were unique to a few individual participants. This helped to build precision, density, and complexity into the emerging theoretical assumptions, and kept them grounded in the data [55, 56]. In the present study, those who had suffered rEPL were reporting divergent narratives to those who reported a singular EPL, therefore theoretical sampling enabled further recruitment of women who had suffered rEPL and the distinct analysis we present below.

### Data collection and management

An interview schedule (see *Supplementary File* 1) was developed in conjunction with the *Petals* and *Ectopic Pregnancy Trust* charities. Interviews were semi-structured [57] and conducted via video-conferencing software, between March and June 2022. Variation in question order and minor alterations to the interview schedule were permitted, to allow for flexibility to pursue pertinent issues raised by previous participants. Interviews lasted between 39 and 159 min (*M*_*Time*_=81 min), were digitally recorded and transcribed verbatim. With respect to data processing, verbatim transcripts were checked against the original audio and any incorrect incidences of transcription were corrected manually by the researchers. Data were then imported into a table in Microsoft Word, where they were subsequently managed and analysed. The interview schedule was annotated during interviews if questions were not received positively or if they were clumsy when spoken as opposed to written. Edits were minor and often stylistic, with no significant change to content (i.e. complete removal or addition of questions) being necessary. Memo notes were taken by hand and added to the bottom of the transcripts after transcription. All participants were assigned a culturally-salient pseudonym by the research team. Full demographics can be found in Tables 2 and 3.

Recruitment ceased when saturation was reached, as measured on two axes: (i) data saturation, where similar data were collected across most of the dataset; and (ii) theoretical saturation, whereby themes were adequately supported by enough data to form a theory.

### Data analysis

Data were analysed by one researcher [FEK-N] using Grounded Theory Analysis [58] appropriate for cross-disciplinary health research [59]. A Classical approach to Grounded Theory Analysis was favoured, due to the pursuance of analytic emergence, and the philosophical rooting of the approach being shared with the research team. Furthermore, given the interviewing researchers [FEK-N, SAS] had no personal experience of pregnancy loss, the idea of a social constructionist (with analytic emphasis on the artefact of the co-created interview) or social constructivist (with analytic emphasis on the co-created interview itself) approach was deemed inappropriate. Data were collected and analysed simultaneously, with no *a priori *assumptions about the topic of interest or the population, and without consultation of the published literature. Analysis was inductive and iterative, with constant comparison between analysed transcripts. Only audio data were used in the analysis.

First, lower-order, line-by-line coding was conducted using verbatim words from the transcripts, to reduce each sentence to a single word or key phrase, to conceptualise core ideas grounded within the text [60]. A second pass of the analysis followed, with higher-order, focused coding undertaken to achieve more conceptual understanding over larger parts of the text [59]. Third, lower-order themes or ‘super-categories’ were developed by merging and re-arranging focused codes, and these super-categories were then sorted, organised, and grouped into higher-order themes; thus, transforming basic data into more abstract concepts and allowing a theory to emerge [61]. Bracketing of preconceived ideas [54] prevented the influence of ongoing and published work from the same team [34, 43, 44] influencing the selection of theme names. Memo notes and wider team members were consulted at this point to ensure no important information had been forgotten or excluded. Trustworthiness and credibility was enhanced by debating the codes, super-categories, themes, and theory, amongst the research team and with members of our Patient and Public Involvement and Engagement [PPIE] group; in the form of defences, as prescribed by Grounded Theory methodology [59].

**Table 2:** Participants’ pregnancy losses and dates

Participant Pseudonym^1^	Pregnancy Losses	Date of Pregnancy
Margaret	Ectopic Pregnancy Early Miscarriage	January 2021 March 2021
Lilly	Early Miscarriage Early Miscarriage Early Miscarriage	June 2020 January 2021 January 2022
Alice	Early Miscarriage Early Miscarriage	June 2020 August 2021
Seniya	Early Miscarriage Early Miscarriage	August 2020 May 2021
Eileen	Ectopic Pregnancy Chemical Pregnancy	February 2021 July 2021
Penelope	Early Miscarriage ToP PUL Early Miscarriage	March 2020 September 2020 March 2021 August/September 2021
Helena	Early Miscarriage Early Miscarriage	July 2020 January/February 2021
Lola	Early Miscarriage Early Miscarriage Early Miscarriage Early Miscarriage	May 2020 June 2020 September 2020 March/April 2021
Emilia	Early Miscarriage Early Miscarriage	October 2020 March 2022
Charlotte	Early Miscarriage Ectopic Pregnancy	April 2021 November 2021
Libby	Missed Miscarriage PUL Chemical Pregnancy	November 2020 February 2021 July 2021
Francesca	Chemical Pregnancy Ectopic Pregnancy Ectopic Pregnancy	December 2020 April 2021 July/August 2021
Ariana	Ectopic Pregnancy Early Miscarriage Ectopic Pregnancy	December 2020 November 2021 April 2022
Kiara	Ectopic Pregnancy Early Miscarriage Early Miscarriage Ectopic Pregnancy	August 2020 January 2021 April 2021 August 2021
Molly	ToP Early Miscarriage	June 2021 January 2022
Scarlett	Early Miscarriage Early Miscarriage Chemical Pregnancy Molar Pregnancy	March 2020 June 2020 November 2020 April 2021

**Notes**: ^1^ Participants were provided with a culturally salient pseudonym by the research team, using the same first initial of their first names

**Table 3:** Participant demographics

Demographics		*N*/16	Percentage of Dataset	Demographics		*N*/16	Percentage of Dataset
**Age (Years)**	18–20	0	0.0	**Gravidity**	Primigravida	5	31.3
	20–24	0	0.0		Multigravida	11	68.8
	25–29	0	0.0				
	30–34	5	31.3	**Planned Pregnancy**	Yes	12	75.0
	35–39	6	37.5		No	4	25.0
	40–44	3	18.8				
	45–49	1	6.3	**Had COVID-19** ^**3**^	Yes	9	56.3
	≥ 50	1	6.3		No	7	43.8
**Geographical Location** ^**1**^	London, England	1	6.3	**Ethnicity** ^**4**^	White (incl. English, Welsh, Scottish, Northern Irish, or British; Irish; Gypsy or Irish Traveller; Roma; and Any other White background)	15	93.8
	North East, England	0	0.0				
	North West, England	2	12.5				
	Yorkshire and the Humber, England	2	12.5				
	East Midlands, England	0	0.0				
	West Midlands, England	0	0.0		Asian or British Asian (incl. Indian; Pakistani; Bangladeshi; Chinese; and Any other Asian background)	0	0.0
	East of England	5	31.3				
	South East, England	2	12.5				
	South West, England	2	12.5				
	Wales	0	0.0				
	Scotland	1	6.3		Black, Black British, Caribbean, or African (incl. Caribbean; African; and Any other Black, Black British, or Caribbean background)	0	0.0
	Northern Ireland	1	6.3				
	Other (incl. islands)	0	0.0				
					Mixed or Multiple Ethnic Groups (incl. White and Black Caribbean; White and Black African; White and Asian; and Any other Mixed or multiple ethnic background)	1	6.3
**Religion** ^**2**^	Christian (incl. all denominations)	4	25.0				
	Roman Catholic	2	12.5				
	Jewish	0	0.0				
	Muslim	0	0.0		Other Ethnic Group (incl. Arab; and Any other ethnic group)	0	0.0
	Hindu	1	6.3				
	Sikh	0	0.0				
	Agnostic	0	0.0	**Marital Status**	Married	9	56.3
	Any Other Religious Belief	0	0.0		Co-habiting	6	37.5
	Atheist or No Religious Belief	9	56.3		Single (incl. Never Married, Divorced, & Widowed)	1	6.3

**Notes**:

^1^ Geographical location based on International Territorial Level – a geocode standard used by the Office of National Statistics in the United Kingdom

^2^ Religion was self-defined. Where participant was non-practising, but gave their religious heritage, the religion was recorded

^3^ COVID-19 diagnosis could have been at any time, not just during pregnancies

^4^ Ethnicity was self-defined by participants and subsequently assigned to the list held by the Office of National Statistics devised for the 2021 Census

## Analysis & results

**Fig. 1:**
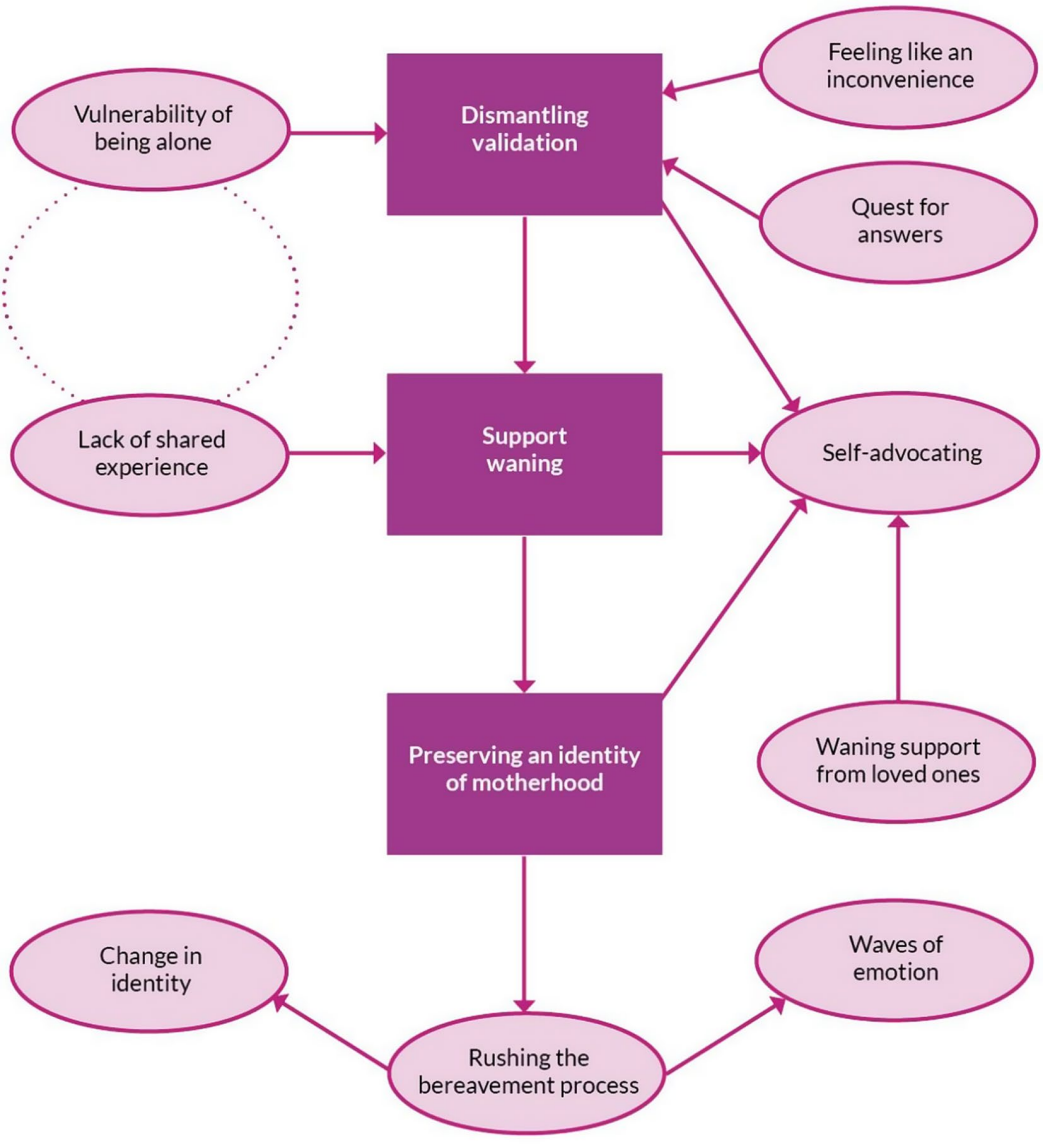
Diagram of super-categories

Analysis resulted in three distinct, but inter-related themes, each comprising several super-categories. Their initial relationship can be seen in Fig. 1.

**Fig. 2:**
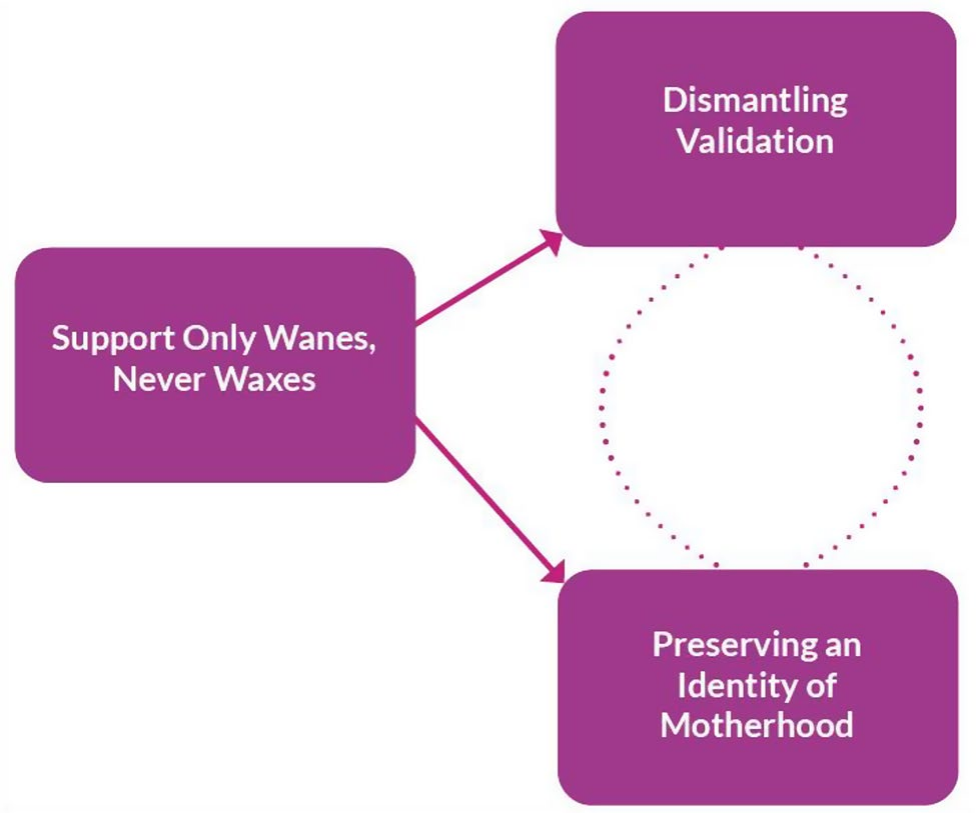
Diagram of Final Themes

The final themes from which our theory was derived are as follows (see also Fig. 2):

- Dismantling Validation.
- Preserving an Identity of Motherhood.
- Support Only Wanes, Never Waxes.

The most illustrative quotations are presented for each theme in the analysis below.

### Theme 1: Dismantling Validation

This first theme explores women’s experience of care for early pregnancy loss during the SARS-CoV-2 pandemic. There was a sense among women that the significance of their loss was invalidated through a lack of empathy and understanding from healthcare professionals, which was exacerbated by a lack of partner presence within the healthcare setting. Some participants felt the pandemic put a significant strain on the healthcare system and thought having an early pregnancy loss was not a significant enough reason to seek out medical attention.

*I didn’t contact anybody*,* in terms of healthcare professionals*,* because I just thought*,* there is nothing that they can do*,* really. I know that I am not far enough along to access the early pregnancy unit. I knew that the GP practices were overwhelmed with other stuff at that time*,* so I just managed things at home by myself.* – **Lola**.

*…I think that*,* I feel that early pregnancy loss was basically like oh*,* that’s just something that you can deal with at home by yourself. You don’t need the NHS to help you with this. This is just a burden on us. This is not something that’s important right now.* – **Libby**.

Although managing the loss alone was a common experience, women discussed the vulnerability of being alone at this time, and many spoke of pregnancy loss being a lonely experience, irrespective of the support available to them:

*But yes*,* I don’t know how to describe the grief. It feels… Even though you have people around you and you talk to people*,* it still feels like a very lonely sort of grief. But I guess all losses you deal with yourself*,* and with others*,* but you have your own thing going on in your head.*– **Ariana**.

These feelings of vulnerability and loneliness were intensified, and the trauma of their experience made worse, by exclusion of partners from the healthcare setting during SARS-CoV-2 restrictions:

*I don’t think that will leave me [becomes upset]. Because it was really difficult being by myself. I don’t think anyone should*,* no one should have to go through what I had to go through but to have to do it by yourself*,* is very difficult.* – **Libby**.

*It was just so traumatic*,* and I felt trauma*,* and thinking about it now*,* I still feel traumatised by it. It was a horrible experience and because of COVID*,* my partner couldn’t come in with me. He’s sat out in the car*,* with my daughter. He can’t come in. I’m having to go through all of this all by myself. And it was utterly terrifying and so distressing* – **Penelope**.

Being alone also left women without an advocate, at a time when they were less able to speak up for themselves, process information, or ask questions:

*…you just felt so isolated*,* there’s nothing you can do. There’s no one there to go and grab somebody for you. There’s nobody there to just talk to you or distract you. You’re just sat there with your thoughts…It’s just horrible.* – **Charlotte**.

*If you zone out or start crying or something and can’t really take in the information*,* it’s quite difficult to retain whatever’s been said.* – **Ariana**.

Finally, for women who did not seek healthcare for their EPL, there was a strangeness to managing this health event on their own:

*I did feel quite strange that you can have the whole miscarriage and not see anyone*,* not see a doctor*,* not have any kind of hospital appointment*,* you’re just sort of left to your own devices. It was very much like*,* Right*,* well*,* at the end of the two weeks*,* I think it’s two weeks after you stop bleeding*,* do a test*,* and if it’s negative then that’s it. That was all the advice I got from the hospital*,* which I just found very strange.* – **Helena**.

Women often reported feeling like an inconvenience, and felt the significance of their loss was invalidated, through a lack of empathy and understanding from healthcare professionals – all exacerbated by a lack of partner presence in the healthcare setting:

*So*,* I think if my husband would have been there*,* I would have got listened to more because he would have kicked up a real stink*,* but because I was on my own and because I was vulnerable and because I just… I just wasn’t in the right frame of mind*,* as you can imagine. I just couldn’t defend myself. I had nothing left… And look*,* I don’t mean to be like he’s my knight in shining armour*,* he needs to come and do that*,* because he absolutely doesn’t*,* but I was in such a vulnerable position that I needed someone to fight my corner* – **Seniya**.

Those who did seek medical attention for their EPL felt that they were an inconvenience to the healthcare staff or a counterfeit within the healthcare setting and maternity healthcare specifically:

*I think my experience when I was admitted on the ward*,* for the medical management…*,* just feeling like I was a bit of an inconvenience to people…And just being left to deal with things myself… I don’t know how to explain it. Not feeling I was a valid patient*,* because I wasn’t on the rest of the ward with all the other people*,* I was just an extra thing that they had to deal with that day.***– Lola**.

*…I found a lot of times it’s like you feel like you’re a second-class citizen the minute you’re not pregnant*,* in lots of different ways. I certainly particularly felt that through the care journey and the fact that almost I was an interloper*,* I was a pregnant person who then became an interloper in a world of the maternity hospital*,* I think.***– Francesca**.

Loneliness and isolation felt by participants led them to seek reassurance from healthcare professionals:

*So*,* I think that acknowledgement from staff*,* ‘No*,* we understand that this is going to be a really difficult time for you*,* we know what is going to happen*,* here are the people you can call on if you need anything. We are just going to be here.’ Just the acknowledgement*,* ‘Even if you think we might be busy*,* please just ring your buzzer or call out.’***– Lola**.

*I felt what she said to me about*,* ‘It’s not really a baby’*,* was quite inappropriate*,* and I think the way that she came up to me and said*,* ‘you’re getting a bit emotional*,* aren’t you?’. I felt*,* again*,* was difficult because I felt that just made me feel on the back foot and it made me feel vulnerable and it made me feel embarrassed and maybe a little bit ashamed about how I had reacted to what was quite a traumatic situation.* – **Francesca**.

Whilst reassurance was rarely forthcoming and some women described being spoken to insensitively, when reassurance was provided, women felt validated:

*But it made the world of difference to me because I felt suddenly now like*,* ‘Oh*,* I’m not a bother. Oh*,* I’m vindicated. It was really bad.’ And as counterintuitive as it is*,* to be told*,* ‘Yes*,* you almost died*,*’ was relaxing to me because I was like*,* ‘I wasn’t going mad.’* – **Eileen**.

Women spoke of their need to find reasons for their EPL and their distress during their quest for answers that these explanations were not forthcoming:

*And annoyingly*,* I’ve not been diagnosed with anything medically. And I say that*,* annoyingly*,* because actually if I’d have been*,* a little bit like the later loss*,* if I had answers*,* something tangible*,* I could do something with that*,* but actually I don’t have anything tangible.* – **Seniya**.

*I find*,* mentally*,* the miscarriage much more difficult than the ectopic. Because the ectopic*,* there was a reason for it*,* they were able to tell me reason*,* they removed the problem*,* I thought that it was plain sailing from there.*– **Margaret**.

This inability to find answers often added to feelings of invalidation, magnified their ability to process the loss, and made them fearful of about the prospects of having a successful pregnancy in the future:

*Because all of our previous ones have just disappeared and we’ve had dismissive comments like*,* ‘It was so early*,*’ ‘You’re so young.’ We’ve been trying for five years now and had nine miscarriages: it’s not like it’s going better and better. It feels good to have an explanation for this one*,* at least*,* and know that there was something there. I mean*,* you gaslight yourself into thinking that maybe it’s not that bad or maybe it’s not…it’s happened but it was so early.* – **Ariana**.

*And*,* like I said*,* I’m now at this stage where I’m waiting to see a specialist to find out what’s going on. But I’m having to wait until August*,* potentially. So*,* I’m now in this complete limbo of what happens if I do get pregnant again between now and then and I miscarry again? Have I just got to accept?* – **Lilly**.

### Theme 2: Preserving an Identity of Motherhood

The second theme explores women’s emotional journey through rEPL, where women reflected on the emotional impact having multiple pregnancy losses had on them as women who had expected to become mothers. Seen here within is the emotional upheaval women felt, and almost an entrapment they face in this search for an identity akin to motherhood, albeit the quest was married by repeated bereavement:

*It is such a roller coaster*,* and you have just got to put that harness on and cling on and just ride it because it does get easier***– Kiara**.

Many women spoke of emotions that came in waves, and a toll that grew larger as the number of losses increased:

*I would always describe grief and that grieving process; it’s like a wave. Because I’m able to say that now*,* and say ‘Actually*,* my life’s pretty good as it is*,* and if we can’t get there*,* we can’t get there’*,* but that’s not to say that this time next week something*,* [laughs] something will trigger me*,* something will tip me over and I’ll be like ‘Oh that’s terrible*,* [laughter] I hate my life*,* I just wanted to have kids.’ So*,* I think it’s very up and down [laughs].***– Scarlett**.

*The whole thing is incredibly hard*,* and I don’t think it ever leaves you and I think each time it happens it chips away a little bit more of you and it does get harder and harder*,* which is why I’ve sort of said after this one I’m done. Regardless of what happens*,* I’m very*,* very fortunate I have got a child.* – **Senyia**.

Women’s strong desire to have a baby and regain an identity of motherhood led many to become fixated with falling pregnant again, often rushing the bereavement process:

*I was desperate. My only concentration in that period afterwards was just about how to get pregnant again… I couldn’t quite function*,* but I was solely focussed on getting pregnant again.* – **Margaret**.

Unlike the joyous feelings they had felt in early pregnancy prior to their loss, participants spoke of subsequent pregnancy as a joyless time, overwhelmed by fear:

*I wasn’t expecting things to happen so quickly. I was just very*,* very nervous. I was terrified. Every time I went to the toilet*,* I was terrified of seeing some blood. I literally found the whole process very nerve-wracking.* – **Charlotte**.

*I was really scared. I was exceptionally scared about the second pregnancy. I had a grief counsellor by that point*,* and I described it as almost like having had a cancer that might kill you and then just injecting the cancer straight back into you. That was how I felt. I didn’t feel overjoyed about the pregnancy because I felt so overwhelmingly scared. So*,* it was a really difficult circumstance.* – **Francesca**.

To protect themselves from the emotional pain they felt after EPL, women spoke of becoming guarded in subsequent pregnancies, which prevented some participants from enjoying the earlier stages of pregnancy:

*I don’t think I mentally can deal with another disappointment in such a short space of time. So*,* the whole time I was early pregnant with this one*,* I was like*,* ‘If I lose this one*,* I’m just going to stop trying because I’m going to just take it as: ‘Be happy with what you’ve got’ whereas I was a bit kind of guarded in emotionally attaching to the idea of being pregnant…. And I was like*,* ‘I can’t do it three times in one year’. So*,* I think*,* yeah*,* it really has ruined particularly the early part of this pregnancy for me because I was so anxious*,* such a nervous wreck.* – **Eileen**.

There was a definite shift in identity acknowledged by women who experienced rEPL, from expectant to bereaved mother, to a bereaved mother of multiple lost babies.

*I describe the first one as almost being like a thing that changes you forever. And then the second loss as being the thing that cements the fact there’s no way back for you almost. This was just two weeks after my 40th birthday*,* so it felt like it was the end*,* really*,* of the journey for me.* – **Francesca**.

Women recalled feeling naïve after their first pregnancy loss, with acknowledgment that pregnancy losses are common and a sense of hope that they would have a successful pregnancy subsequently:

*I think after that initial loss*,* there is a little bit of hope of thinking*,* ‘Well*,* it’s okay. We can try again. We’ve got through this.’ But then*,* the reality of…what that looks like is quite difficult.***– Lola**.

When subsequent pregnancy losses occurred, some women recalled feeling resentful towards their bodies and the prospect of a life without children, and a permanent shift in identity:

*And it’s ultimately what we’re on this earth to do: we’re here to reproduce… And then you fall pregnant and it’s like oh there we go*,* that’s my time now*,* and then it doesn’t happen. And then you fall pregnant again and you get so close to it happening and being a happy ending and then that gets taken away again. And the more that happens*,* the less hope you have for that as your future.* – **Lilly**.

### Theme 3: Support Only Wanes, Never Waxes

The final theme addresses women’s experiences of support during, between, and after their rEPLs. There was a strong feeling among participants with rEPL that support waned as the number of EPLs increased. Firstly, women commented on their lack of shared experience. Although women’s partners appeared central to their ongoing support system, many women spoke of their partners feeling disconnected from the EPLs due to the lack of a shared experience:

*So*,* with the first baby I lost*,* <partner’s name>*,* I think he said he almost felt like it didn’t happen because he wasn’t in the room when I was told*,* he couldn’t come to any appointments with me*,* he said he felt quite disconnected from it…* – **Alice**.

Some women felt that they were on a different grieving pathway from their partner:

*This is a very triggering week or two for me. I’ve got a mind for dates*,* which is not handy in some ways. So*,* I knew that Thursday was the day exactly last year at teatime that we got that phone call. Tomorrow is exactly the day since I went and took that medication to start that TfMR. I know how long it is since we miscarried*,* he doesn’t necessarily.***– Molly**.

Although women felt they needed an increasing amount of support as the number of EPLs increased, participants discussed how support from loved ones often waned over time:

*And it doesn’t always help to hear ‘oh*,* it’s just one of those things’; I just need somebody to say ‘you’ll be alright*,* it will take time*,* but you’ll be alright. We’re here to help you get through this’.* – **Libby**.

*The first ectopic*,* we had flowers and presents and people visiting. It was lovely. The second one*,* we still did a bit. The third*,* a bit less and the fourth*,* it trailed off by then.***– Kiara**.

The waning support was a defining feature of many women’s rEPL experiences, with women reporting how they felt they were being defined by their rEPL status:

*…I don’t know if that was COVID related*,* or just because it is the third time now*,* and it is almost… I have definitely noticed a shift in how people approach that. It is almost like [laughing]: ‘Well*,* you keep trying*,* so you keep putting yourself through this’. No-one has said that*,* and I don’t know if that is just what I am interpreting*,* maybe*,* but yes.***– Emilia**.

*Yes*,* it’s been really tough……… [Crying] ………And I think I thought the first one was an anomaly*,* so the second one was quite tough*,* and you get*,* if you like*,* [sniffs] either less people offer support or they don’t offer the same level of support after the first*,* yes*,* after one*,* they kind of think oh that just happens to <participant’s name> now. Almost like you’ve got a disease*,* in a way.***– Alice**.

Despite this, there was a strong desire for support and empathy from loved ones, but a recognition that they often lacked an understanding of how to help, and often made unintentionally hurtful comments :

*…for some people*,* I think they look at baby loss*,* especially if it’s only early days of a pregnancy*,* it doesn’t really matter. Again*,* it’s a foetus*,* a bundle of cells*,* that’s what you see it referred to. Actually*,* it’s not*,* it was my baby*,* and <Baby Name> really was a baby. We held her*,* we saw her*,* we felt her. With the miscarriage*,* I didn’t have that experience*,* and I think in some ways I found that harder. I got to say goodbye to <Baby Name>*,* where this baby I didn’t*,* I didn’t have that closure*,* almost*,* I didn’t feel I was allowed to feel that loss or grief process…***– Molly**.

*So*,* I think a lot of people do try to brush it off or you get the whole*,* ‘At least you can get pregnant*,* at least it was early on.’ My mum came out with a blinder*,* bless her*,* she said*,* ‘It was like when I lost my cat.’ And I was like*,* ‘No*,* mum*,* it’s not like losing your cat at all.’* – **Helena**.

A lack of understanding was particularly evident when women suffered EPL after IVF:

*The last one*,* I guess because it was a bit earlier*,* my mum sort of said to me*,* ‘Oh it’s okay*,* you can try again*,*’ and honestly*,* that*,* especially after having had IVF as well*,* because it’s not just the getting pregnant bit*,* it’s the bit before that you have to go through as well. So yes*,* so things like that*,* ‘you can try again’ or ‘it’s okay*,* it just wasn’t meant to be*,*’ just those sorts of things are just super unhelpful*,* and a lot of people just don’t know what to say.* – **Seniya**.

A lack of support from loved ones promoted women’s self-advocacy, regarded by some as fundamental to getting through such an arduous time:

*I think the only other piece of advice I think I could give is to stay strong and don’t feel ashamed of how you feel*,* if that means that you have to unfollow people on Instagram or to not go to baby showers or to not deal with that*,* you have to put your own life jacket on first and all of this in order to get through what is a really*,* really stormy sea.* – **Francesca**.

Some women emphasised that there were on an individual grief journey, and had to adapt their lives to cope, rather than expect others around them to respect or understand their needs.

*It depends who it is. I think some people*,* very poorly. Like that comment I said about: ‘This is the…’ I have loads of comments*,* especially the third time around. ‘You know you need to stop trying now. You can’t keep putting yourself through this’*,* that kind of thing. I have got one or two. I think the people that have been through it are probably the most supportive. But I have got friends that haven’t*,* and they have been very supportive*,* just in terms of listening and being there*,* really. [pause] Yes. I just think it depends on the person.***– Emilia**.

At times, coping involved secrecy, as women found it increasingly difficult to tell loved ones that they had lost another baby:

*So*,* I think with the second one*,* I don’t know if we’d told anyone yet. I think I told my mum. [Pause] Yes*,* I did*,* I told my mum; I was walking home from the hospital*,* and I rang her coming out of the hospital. It’s really interesting because I think people underestimate the impact that it has on your other relationships as well*,* pregnancy loss.* – **Scarlett**.

Adapting meant avoiding triggering situations, particularly being around children. Many commented on feeling resentful towards friends and family making pregnancy announcements, even when they themselves had a successful pregnancy:

*The situations I put myself in*,* I really think about*,* whether I feel able to do that and how I feel with the person.* – **Alice**.

*Well*,* feeling jealous of my friends. The pregnancy announcements*,* the celebrity pregnancy announcements*,* being jealous of just anybody who has managed to achieve*,* to have more than one child. It’s crazy*,* isn’t it?***– Libby**.

Interestingly, the SARS-CoV-2 lockdown restrictions were sometimes helpful to women needing to distance themselves from certain individuals or situations, but exacerbated loneliness in others:

*I felt quite relieved that it was a time when we were all working from home*,* so I didn’t need to see people. I think I was restricted*,* in how nobody could visit us*,* so I was protected in a sense*,* [laughing] that I didn’t have to see people.***– Lola**.

…*so*,* it was almost a blessing and a curse*,* if you see what I mean. In some ways*,* it was easier to disconnect telling people*,* not in a face-to-face environment but at the same time it’s quite lonely*,* it’s very lonely actually*,* and actually having to battle through everything that I went through with that*,* that was lonely.* – **Seniya**.

### Interpretation of the theory: ‘Knights in Shining Armour and (M)others in Life Jackets’

Grounded Theory Analysis elicited three main themes in relation to women’s experience of rEPL during the SARS-CoV-2 pandemic: Dismantling Validation; Preserving an Identity of Motherhood; and Support Only Wanes, Never Waxes. The way in which these themes give rise and relate to one another allows for the emergence of the theory itself: *‘Knights in Shining Armour and (M)others in Life Jackets’* (see Fig. 2). The theory describes women’s experience of being ‘othered’ in maternity healthcare settings and in wider familial, friendship, and societal circles. Women felt their experiences of rEPL were seen as unusual, and seemed unapproachable as a topic of discussion – a social taboo. Women expressed the necessity to advocate for care and acknowledge their status as a mother (albeit bereaved), alone. The relationship was cyclical: the greater the perceived lack of validation about rEPL, the greater the desire to fight for the preservation of a motherhood identity, and vice versa, albeit that these relationships themselves presented with variable strength (as shown by the dotted, rather than solid lines). Affecting both of these experiences was the constant perception that support was only ever waning with each subsequent pregnancy loss – further othering these women from mothers of live infants in the eyes of those around them, and – painfully – also through their own gaze as mirrored back to them. Throughout all themes were two common principles: the effect of the lack of partner presence, and the notion of having to advocate for oneself. Whilst women did not need a ‘Knight in Shining Armour’ to protect them, having partner, societal, and professional support could have alleviated some of the strain that they faced as they ‘Put on Their Own Life Jacket’ to try to understand their medical condition, and improve their psychological and physical wellbeing.

## Discussion

### Summary of main findings

This study provides new insight into the previously unexplored experiences of women who suffered from rEPL during the SARS-CoV-2 pandemic. This study affirms these recent findings while also magnifying systemic issues with EPL care already present prior to the pandemic [15]. Undeniably, there were unique circumstances and healthcare policies during this time contributing to isolation and exacerbated feelings of loneliness and despair during the bereavement journey. This study demonstrates navigating rEPL during these turbulent times was an extremely challenging and traumatic experience. A lack of support from partners, friends, and family, as well as HCPs above all, obliged bereaved mothers to self-advocate to protect both their mental wellbeing and re-establish their desired motherhood identity. At the same time, the significance of their loss was dismantled and invalidated by a poor societal understanding of their arduous journey and by a general lack of empathy. With repeated cases of loss, women had an even stronger need for societal and professional backing; however, support and empathy waned gradually with every instance of loss.

Lack of support for bereaved mothers was not limited to professional healthcare environments. Inadequate and diminishing support from loved ones further invalidated women’s societal and personal perception of their loss which propelled many women further down a lonely and isolated bereavement pathway. Prior research has shown that women commonly feel that there is a general lack of psycho-social support from friends and family following a loss [62, 63]. This study supports these findings while further proposing that, in cases of repeated pregnancy loss, support waned gradually with every instance of loss. This latter observation is not consistent with data collected prior to the pandemic [64, 65]. Many women believed this was due to a lack of understanding of the nature and magnitude of grief and how to empathetically address their trauma.

Another observation that is unique to rEPL is the fixation with becoming pregnant again shortly following a loss. This appears to stem from a strong desire to re-establish the motherhood identity to which the bereaved had grown accustomed. An exacerbating factor was the participants’ advanced average age (majority over 35), and a consequent difficulty to cope with the knowledge of their declining fertility. Debate continues within the literature regarding the optimum interpregnancy interval following a non-molar or non-ectopic pregnancy loss [66], with suggestions on the waiting period ranging from less than three months to at least six months. Whilst from a physiological standpoint, evidence suggests that a short inter-pregnancy interval after pregnancy loss is associated with a lower risk of subsequent miscarriage [66], this recommendation does not consider the psychological consequences of rushing the bereavement process.

This study demonstrates that women strongly favoured a shorter inter-pregnancy interval during the pandemic, which clearly had adverse consequences on the mother’s psychological well-being during subsequent pregnancies, whilst also putting pressure of their relationships – particularly the one with their partner. Consistent with findings predating the pandemic, participants described the early stages of a new pregnancy following loss to not be joyful but a time of heightened anxiety and bracing for potential future loss [22, 67]. To protect themselves, women implemented coping strategies, such as dissociating from the state of pregnancy or a possible future with their unborn child.

### Strengths, limitations, and future directions

To our knowledge, this is the first qualitative study to look at women’s experience of rEPL in the UK during the SARS-CoV-2 pandemic. The rigorous nature of the methodological approach utilised in this study and the use of semi-structured interviews allowed for women to provide a direct narrative report of their experiences free from preconceived ideas concerning their beliefs and feelings about the topic discussed. This allowed for an in-depth insight into the psychological, social, and community factors which may have impacted bereaved mothers’ experiences. Furthermore, the use of video-conferencing facilitated nationwide participation, resulting in a regionally diverse representation of women cared for by various NHS trusts, thus improving the transferability of findings. Another major strength was the cross-disciplinary nature of the research team, which enables findings to be relevant to theory and practice in the social, psychological, and health sciences. Findings from this study have highlighted both pandemic-specific shortcomings and more systemic issues, which have potential applications in revising policy and practice for EPL care.

We acknowledge the limitations to the transferability and generalisation of the data collected; as self-selection can result in an unusual sample – such as ours which is representative of an older population, who may not necessarily representative of population of pregnant women in general. We note there is an over-representation of women with ectopic pregnancy [68], which may have affected the study outcomes and recommendations. Also, although the conducting of virtual interviews was necessary for data collection, it may also have inhibited some participants from open discussion where they found it challenging to establish a rapport from a distance. More importantly, however, we acknowledge the lack of ethnic diversity among our participants, despite our efforts to recruit a representative sample. This issue has also been identified by previous studies [40, 43, 69]. Whilst the under-representation of certain rarer EPL typologies, such as molar pregnancy whose unique pathophysiology requires special treatment and leads to different outcomes, can be seen as a limitation, this study performed similarly to previous studies recruiting EPL populations [15].

Furthermore, our study contributes to the extant literature-base [70–76] suggesting there remains a dire need for evidence-based psycho-social interventions for women who suffer EPL, and especially rEPL. As ever, new studies highlight specific areas which require further exploration and future research should pay careful attention to the recruitment of a more ethnically diverse group of women, as well as other often understudied populations, including those who experience rEPL and are: unmarried or not co-habiting, lesbian, gay, bisexual, non-binary, or transgender, those experiencing severe mental illness, or living with high levels of social complexity. Those with rarer forms of EPL require specific empirical attention, as do partners of women who suffer rEPL. The way in which to more successfully engage with these suggested groups may be through more creative methods, including, but not limited to arts-based approaches, diary studies, photo-voice, and shared blogging or messaging-based support groups.

## Conclusion

This study demonstrated the enduring psycho-social effects of rEPL during the SARS-CoV-2 pandemic. Reported as an extremely isolating and traumatic experience, often the negative and progressive feelings associated with rEPL were profoundly exacerbated by a lack of professional and societal support. This study brings to light the need for psycho-social support to grieving mothers, in the aftermath of pregnancy loss, during the inter-pregnancy period, and throughout subsequent pregnancies. While the lack of empathy and aftercare by both healthcare professionals and society at large is the most prominent observation from the latter subset, the single greatest pandemic-specific factor is the absence of partners from the healthcare environment. As compared to patients suffering later-term loss, there was a complete and utter lack of leniency on this policy. This arbitrary two-tiered approach reasserts prior findings that early instances of pregnancy loss and their devastating psychological consequences are often underappreciated or overlooked. The move to blanket changes to healthcare policy in response to the pandemic health system shock was a mistaken one, especially when it came to policy surrounding EPL.

Women who have suffered an EPL often feel marginalised and that the significance of their loss is overlooked by society. By offering a lens through which participants can voice their own narrative, women’s anecdotes are brought to the foreground. This, in turn, can challenge the longstanding-imposed views of EPL, brought about through traditional hegemonic and masculinist discourses. Drawing from the compelling narratives of the strong women who participated in this study, we must advocate unequivocally for competent and empathic approaches to improving the care for mothers suffering from such a grave rupture in their lifecourse.

## Electronic supplementary material

Below is the link to the electronic supplementary material.

Supplementary Material 1

## Data Availability

The data supporting the findings of this study are available upon reasonable request from the corresponding author. The data are not publicly available due to privacy or ethical restrictions. The interview schedule is available in the supplementary materials associated with this article.

## Declarations

### Patient and Public Involvement and Engagement

This work was formed as a sub-study of The PUDDLES Study, which has looked at the experiences of late-miscarriage, stillbirth, neonatal death, and associated care during the SARS-CoV-2 Pandemic. PUDDLES is one of many UK-based studies which feeds directly into PIVOT-AL. At a meeting of PIVOT-AL in November 2021, two areas of research were identified as being unaddressed by current or ongoing pandemic-related portfolios of research: early pregnancy loss and early elective abortion care. This study was therefore devised in response to a call from PIVOT-AL for researchers to plug this lacuna. The PUDDLES programme of work has been discussed with members of the NIHR ARC South London Patient and Public Involvement and Engagement [PPIE] meeting for Maternity and Perinatal Mental Health Research (July 2020; June 2021), which has a focus on co-morbidities, inequalities, and maternal ethnicity; a meeting with the Chief Midwifery Officer and the Maternity Transformation Team of NHS England and NHS Improvement (July 2020), at a meeting focused on multi-morbidities and maternity safety; and an NIHR ARC South London Work in Progress Meeting (October 2020), focusing on maternity and perinatal mental health research. This work has also been discussed at PIVOT-AL national collaborative meetings (November 2021; April 2022; September 2022), a research collaborative which is leading on the national response for policy makers during the pandemic; and to NHS England and Improvement’s Chief Midwifery Office (December 2021), which focused on early insights from new research on maternity services to inform service COVID-19 recovery. Further input was received PPIE members at King’s College London’s Department of Women & Children’s Health Maternal and Perinatal Mental Health Systems & Policy Research Group Meeting (October 2021) and the Department’s PPIE Group for Perinatal Bereavement, Trauma, & Loss (March, June, & October 2022). To ensure sensitivity and appropriateness of recruitment materials and interview schedules, and to aid recruitment, The PUDDLES Study team originally worked with the International Stillbirth Alliance, Tommy’s Charity, and Sands. Subsequently, PUDDLES – Early Pregnancy Loss has worked in conjunction with Petals: The Baby Loss Counselling Charity, The Ectopic Pregnancy Trust, and The British Pregnancy Advisory Service. Through all these engagements, we received feedback on recruitment, study design, and interpretation on findings from both lay and expert stakeholders, including members of the public, those with lived experience, health and social care professionals, researchers, and policy makers.
