# Supplementary material for: Knights in shining armour and (M)others in life jackets: Women’s experiences of advocating for care alone when suffering recurrent early pregnancy loss during the SARS-CoV-2 pandemic
Source: BMC Public Health. 2025 Jan 29;25:367. doi: 10.1186/s12889-024-20882-2 (PMC11776267; doi:10.1186/s12889-024-20882-2)
Supplement: Supplementary file 1 — Supplementary Material 1 [file 12889_2024_20882_MOESM1_ESM.pdf]

# – PUDDLES: Early Pregnancy Loss Study Interview Schedule –

Early Pregnancy Loss and The Pandemic

Ethics Reference Number:- HR/DP-21/22-28808

Thankyou for taking the time to be interviewed in relation to your recent experiences of pregnancy loss.

Introductions: You provided your details to us and have agreed to talk to us in a little bit more depth about your experiences, and we are really grateful for you agreeing to do so. What I'd like to do in this interview is to ask you about your experiences of your pregnancy loss, in a bit more detail, and for you to tell me, in your own words, what happened including anything in relation to your experiences in the context of the COVID-19 pandemic.

There are no right or wrong answers – we are interested in your own experiences, perspectives, feelings, and opinions. We can take as long as you need to talk. We can pause or stop altogether at any time.

There are three main parts to today's interview; the first is about the pregnancy loss experience itself; the second is about your bereavement and grieving process; and the final section is about care during and after pregnancy loss in general.

Do you have any questions before we begin? If not, I shall now start to record.

Demographics:

Can I just ask you a few quick demographic questions and some more informational questions about your pregnancy?

- Age
- Marital status?
- Ethnicity?
- Religious faith, if you have one?
- Was this your first pregnancy?
    - If no, how many times have you been pregnant before and how many children do you have?
- Was this a planned pregnancy?
    - Conceived naturally or via ART?
- When did you find out that you were pregnant?
- In which region of the country do you live?
    - And did you receive care in the same region as the one in which you live?
- Have you or anyone in your household had COVID?
    - Was this at the time of your pregnancy loss?

Opener:

Can I first confirm what type of early pregnancy loss (up to 14 weeks' of gestation) you had?:

- EARLY MISCARRIAGE - PREGNANCY OF UNKNOWN LOCATION -

- ECTOPIC PREGNANCY - MOLAR PREGNANCY - EARLY ELECTIVE ABORTION -

People discuss pregnancy loss in different ways. I would like to use language that you feel comfortable with and so an important question is how do you refer to your pregnancy loss? [***N/B.*** Use THEIR language throughout – which may be 'pregnancy loss'; the specific type of pregnancy loss; or in fact the 'baby'].

Experience of pregnancy loss:

In your own time, and in your own words, could you tell me about your experiences of pregnancy loss during the pandemic?

[**N/B.** Only if no response, use: *Would you like to begin by telling me about your pregnancy?*]

Probes to cover experience, thoughts, feelings, and reactions (If unanswered by first monologue):

- What was your pregnancy like – any issues?
- What was your relationship with your HCP like?
- Was your pregnancy experience affected by COVID-19 lockdown restrictions?
- What was that experience like? How did you feel? Physically? Emotionally?
- Were you at home or in a healthcare setting?
- When did you realise you might have lost the pregnancy or when were you told that you had? Who told you?
- Could you tell me about these conversations – appropriate/sensitive?

How have you communicated your pregnancy loss to friends, family, and other loved ones?

Probes (If unanswered by monologue):

- Who were the first people you told? How have people reacted to your loss?
- [If applicable] How have you communicated your pregnancy loss to your employer?

Have you felt people have understood your circumstances?

Is there anything that you would like to add that I haven't asked you about your loss whether about the experience itself or conversations with others?

That concludes the first part of the interview and we will now be moving on to the grieving process since your experience. Before we talk about that, would you like to take a break or are you ok to continue?

Bereavement & Grieving:

As mentioned, I'd now like to ask you about the time since your pregnancy loss until now.

How have you been? Could you tell me about your thoughts and feelings throughout and since your experience of pregnancy loss?

Could you tell me about any support you have received in the time since your pregnancy loss?

This could be support from healthcare professionals, family, charities, or specialist bereavement/grief counselling services – please go into as much detail as you would like.

Probes to cover experience, thoughts, feelings, and reactions (If unanswered by monologue):

- Who has provided the main source of support?
- What support has been the most helpful? Emotional? Practical?
- What were the things that helped? What didn't help?
- Do you think that the COVID-19 restrictions affect you accessing any services or support?
- How would you have liked to have been supported?
- Has your experience of loss, in your opinion, impacted any of your relationships and if so could you tell me how?
- Is there anything that you would like to add that I haven't asked you about your grieving and bereavement process?

That concludes the second part of the interview and we will now be moving on to the wider topic of pregnancy loss care. Before we talk about that, would you like to take a break or are you ok to continue?

Advice Sharing:

So, these final questions are about your thoughts on care for pregnancy loss more generally.

When thinking about the care you received, what would you change, if anything and why?

Probes (If unanswered by monologue):

- What would be the most important change in your opinion and why?

Was there anything which was done particularly well during the course of your care and why did this make a difference in your view?

Was there anything that could have been improved during your care? How would it have helped?

Probes (If unanswered by monologue):

- What was the most important thing which helped you during this time and why?
- Was there anything that hindered or did not help you during this time? How did it/they impact? [Note: do these to some extent duplicate above and if so may not be needed?]

How would you like to see pregnancy loss discussed and researched in the future and what impact could that have in your view?

And finally, do you have any advice for women who have had similar experiences to yourself?

I have come to the end of my interview questions, but is there anything at all that you would like to add that perhaps I haven't asked you about? Or do you have any questions for me?

If not, thank you very much for your time and again, and should you require any further support, the resource list has links to charities and helplines which are still functioning during the pandemic.

Please do remember to give yourself a bit of time and space after we end this call before you go on to your next activities.
